# Eating Fast Leads to Obesity: Findings Based on Self-administered Questionnaires among Middle-aged Japanese Men and Women

**DOI:** 10.2188/jea.16.117

**Published:** 2006-05-19

**Authors:** Rei Otsuka, Koji Tamakoshi, Hiroshi Yatsuya, Chiyoe Murata, Atsushi Sekiya, Keiko Wada, Hui Ming Zhang, Kunihiro Matsushita, Kaichiro Sugiura, Seiko Takefuji, Pei OuYang, Nobue Nagasawa, Takaaki Kondo, Satoshi Sasaki, Hideaki Toyoshima

**Affiliations:** 1 Department of Public Health/ Health Information Dynamics, Field of Social Life Science, Nagoya University Graduate School of Medicine.; 2 Department of Cardiology, Nagoya University Graduate School of Medicine.; 3 Department of Metabolic Diseases, Nagoya University Graduate School of Medicine.; 4 Department of Food and Nutrition, Faculty of Human Life, Jumonji University.; 5 Department of Medical Technology, Nagoya University School of Health Sciences.; 6 National Institute of Health and Nutrition.

**Keywords:** Eating, Obesity, Body Mass Index, Japan, Cross-Sectional Studies

## Abstract

**BACKGROUND:**

Few epidemiologic studies have examined the association between the rate of eating and obesity. In this study, we cross-sectionally examined the association of the self-reported rate of eating with current Body Mass Index (BMI), and BMI-change from 20 years of age to the current age.

**METHODS:**

Subjects were 3737 male (mean age ± standard deviation and mean BMI ± standard deviation: 48.2 ± 7.1 years and 23.3 ± 2.7 kg/m^2^) and 1005 female (46.3 ± 7.0 years and 21.8 ± 2.8 kg/m^2^) Japanese civil servants. We measured self-reported categorical rate of eating, current BMI, BMI at age 20, and BMI-change from age 20. Energy intake was assessed over a 1-month period with a brief-type diet history questionnaire.

**RESULTS:**

The multiple regression analysis in which the current BMI was regressed by categorical rate of eating, energy intake, age, and lifestyle factors showed that current BMI steadily increased by -0.99, -0.67, 0.81, and 1.47 kg/m^2^ along with the progress of categorical rate of eating from the ‘medium’ group to ‘very slow’, ‘relatively slow’, ‘relatively fast’, and ‘very fast’ groups, respectively, in men. In women, the corresponding values were -1.06, -0.35, 0.50, and 1.34 kg/m^2^. When the BMI increment from age 20 to current age was regressed in the same manner, the increment was -0.63, -0.34, 0.57, and 1.05 kg/m^2^ in men and -0.71, -0.32, 0.34, and 1.14 kg/m^2^ in women, respectively. Additionally, both BMI at age 20 and current height were positively associated with rate of eating.

**CONCLUSIONS:**

Our results among middle-aged men and women suggest that eating fast would lead to obesity.

Obesity is an epidemic in much of the developed world, for example in the United States, with 20% of males and 25% of females now classified as obese (Body Mass Index (BMI) ≥30.0 kg/m^2^).^1^ On the other hand, in Japan, the prevalence of preobese (BMI: 25.0-29.9 kg/m^2^) and obese (BMI ≥30.0 kg/m^2^) men increased from 14.5% and 0.8%, respectively, in the time-period 1976-80 to 20.5% and 2.01% during 1991-95, and obesity has increased gradually among Japanese people, who are generally shorter and leaner than occidentals.^2^ Obesity is currently a major contributor to a range of metabolic disorders responsible for much of the morbidity and mortality that burden Japanese society as well as other developed countries.^3^^,^^4^ The majority of well-designed large epidemiologic studies in Japan have shown that obesity, as indicated by a high BMI, is associated with an increased risk of diabetes mellitus, hypertension, and coronary heart disease, and consequently with increased mortality rates.^5^^,^^6^^,^^7^ Therefore, it is important to understand more about this problem and to provide measures that will lead to both the prevention and correction of obesity.

Eating behaviors, particularly the rate of eating, have long been of interest as one of the factors which contribute to the development of obesity.^8^ Although several studies have explored potential associations, the results have been inconsistent.^9^^-^^18^ To our knowledge, most previous studies on the rate of eating have compared it between obese and non-obese subjects^14^ or have observed weight change caused by decreasing the rate of eating among obese subjects.^13^ Also, study subjects were children,^11^^,^^14^^,^^15^ teenagers,^8^^,^^16^ adults with severe obesity,^13^^,^^17^ or those with metabolic disorders including diabetes and dyslipidemia.^12^ Few studies have examined the association between the rate of eating and obesity epidemiologically in substantially healthy middle-aged adults.^18^ One aim of this study is to obtain more knowledge about the association of the rate of eating with current BMI in Japanese healthy males and females of middle age.

It is not known when the rate of eating is acquired as an eating behavior in life. A person who had rapid eating behavior in childhood may continue to eat rapidly through adulthood. Spiegel et al. reported that it was difficult to maintain a slower rate of eating in the behavioral treatment of obesity.^13^ On the basis of the hypothesis that individuals keep their rate of eating for a long time, we also examined the relationship between the rate of eating and BMI at age 20 years, BMI-change from age 20 to current age, and height that would be affected by nutritional condition till puberty. We propose that the rate of eating is a potentially significant key factor among eating behaviors in obesity prevention strategy and public health practice.

## METHODS

### Study Population and Questionnaire

A survey was conducted for civil servants in Aichi Prefecture, central Japan in 2002. The participants consisted of 5179 male and 1472 female workers aged 35-69 years. The survey included a self-administered questionnaire, a physical examination including height and weight, and collection of fasting blood samples. The questionnaire consisted of two parts. The first part addressed lifestyle characteristics, such as physical activity, smoking status, and alcohol drinking habits. The second part included a brief-type diet history questionnaire (BDHQ)^19^ that required recall of dietary habits over 1 month. The rate of eating was self-reported by an answer chosen from five qualitative categories, that is, ‘very slow’, ‘relatively slow’, ‘medium’, ‘relatively fast’, and ‘very fast’ to the question: ‘How fast is your rate of eating?’. Energy intake (kcal/day) was calculated from the data obtained from BDHQ using the ad-hoc program developed for nutrient calculation of BDHQ.

Weight and height were measured under a fasting state to the nearest 0.1 kg and 0.1 cm, respectively, with subjects wearing light clothing and no shoes. BMI was calculated as weight in kilograms divided by the square of height in meters. Weight at age 20 was obtained from the following open-ended question: ‘What was your usual weight at age 20 years?’ BMI at age 20 was also computed as the weight at age 20 in kilograms divided by the current height measured in square meters. Furthermore, we calculated the BMI-change (kg/m^2^) by subtracting the BMI at age 20 from the current BMI.

We excluded subjects with missing values or subjects with a medical history of cancer, and all variables used in this analysis were obtained from 3737 males and 1005 females. Additionally, we performed the same analysis among only non-diabetics (3433 men and 963 women) to eliminate the effect of diabetic medication by excluding subjects with a medical history of diabetes.

All subjects signed an informed consent statement, and this study was approved by the Ethics Committee of Nagoya University Graduate School of Medicine, Nagoya, Japan.

### Statistical Analysis

All analyses were performed separately for each sex. One-way analysis of variance with polynomial contrast in the SPSS^®^ statistical package was used to evaluate the statistical differences in the rate of eating categories. A polynomial contrast subcommand estimated the linear trends across all categories. The differences in the proportion of answers for categorical variables across the rate of eating categories were assessed by chi-squared test.

To estimate the contribution of the rate of eating to current BMI, we calculated partial regression coefficients and 95% confidence intervals for the rate of eating by multiple regression analysis with the current BMI as a dependent variable. The partial regression coefficients for the respective categories expressing the rate of eating, which were dummy variables with ‘medium’ as the reference, were estimated in three models; Model 1: adjusted for age; Model 2: adjusted for age and energy intake; Model 3: adjusted for age, energy intake, smoking status (never, former, current: dummy variables), the frequency of alcohol consumption per week (continuous), and regular exercise (one day or more per month with the total exercise time of 60 minutes or more per month: Yes or No). Furthermore, we performed multiple regression analysis treating BMI-change as a dependent variable. The partial regression coefficients for the categorical rate of eating were estimated in four models; Model 1: adjusted for age; Model 2: adjusted for age and BMI at age 20 (continuous); Model 3: adjusted for variables in Model 2 plus energy intake; Model 4: adjusted for variables in Model 3 plus smoking status, the frequency of alcohol consumption per week and regular exercise.

All statistical analysis was performed with the SPSS^®^ statistical package for Windows version 11.5. All reported p-values were two-sided and a p-value of less than 0.05 was considered statistically significant.

## RESULTS

The means (standard deviations) of age and current BMI were 48.2 (7.0) years and 23.3 kg/m^2^, respectively, in men, and 46.3 (7.1) years and 21.8 kg/m^2^ in women.

Table 1 shows the anthropometric indices, lifestyle factors and energy intake per day by the rate of eating. The distribution of the rate of eating differed significantly between men and women (p=0.001). The category occupied by the largest number was ‘relatively fast’ (1400/3737, 37.5%) in men and ‘medium’ (425/1005, 42.3%) in women. In men, the mean age became younger as the categorical rate of eating became faster, but this was not true for women. The associations of the rate of eating with anthropometric indices were similar in men and women except the association with body height. Current weight and BMI increased significantly along with the increase in the rate of eating. Current height also showed a significant increasing relationship with the increase in the rate of eating only in men, and this relationship was similar also in women though not significant. Both weight and BMI at age 20 also showed the increasing trend. In addition, BMI-change from age 20 to the current age showed the increasing trend across the rate of eating categories. Energy intake per day also increased significantly with the increase in the rate of eating in men.

**Table 1.** Anthropometric indices, lifestyle factors and energy intake per day by rate of eating.

	Rate of eating categories					P-value	Trend p

	Very slow	Relatively slow	Medium	Relatively fast	Very fast		

	Men (n=3737)						
n (%)	63 (1.7)	395 (10.6)	1383 (37.0)	1400 (37.5)	496 (13.3)		
Age (year)*	49.3±7.3	48.8±7.3	48.5±7.2	47.9±7.0	47.2±7.0	0.01^†^	0.03
Current body height (cm)*	167.7±5.5	168.1±6.0	168.3±5.6	169.0±5.6	169.5±5.6	<0.001^†^	0.01
Current body weight (kg)*	61.5±8.3	62.7±8.1	64.8±8.6	67.7±8.7	70.1±9.4	<0.001^†^	<0.001
Current BMI (kg/m^2^)*	21.8±2.5	22.2±2.5	22.8±2.6	23.7±2.7	24.4±2.9	<0.001^†^	<0.001
Weight at age 20 (kg)*	55.8±6.3	56.1±6.1	57.8±6.9	59.5±7.8	60.8±8.6	<0.001^†^	<0.001
BMI at age 20 (kg/m^2^)*	19.8±2.0	19.8±1.7	20.4±2.1	20.8±2.4	21.1±2.7	<0.001^†^	<0.001
BMI-change (kg/m^2^)*	2.0±2.0	2.3±2.2	2.4±2.3	2.9±2.5	3.3±2.8	<0.001^†^	<0.001
Physical activity							
≥ 60 min/month	39 (61.9)	236 (59.7)	843 (61.0)	805 (57.5)	267 (53.8)	0.06^‡^	
< 60 min/month	24 (38.1)	159 (40.3)	540 (39.0)	595 (42.5)	229 (46.2)		
Smoking status							
Current smoker	26 (41.3)	129 (32.7)	483 (34.9)	497 (35.5)	192 (38.7)	0.23^‡^	
Former smoker	12 (19.0)	105 (26.6)	397 (28.7)	407 (29.1)	144 (29.0)		
Never smoker	25 (39.7)	161 (40.8)	503 (36.4)	496 (35.4)	160 (32.3)		
Alcohol drinking habit (times/week)*	4.0±2.9	3.8±2.9	3.9±2.9	3.8±2.8	3.6±2.9	0.23^†^	0.34
Energy intake (1000 kcal/day)*	2.09±0.62	2.08±0.60	2.10±0.63	2.15±0.68	2.24±0.77	<0.001^†^	0.04

	Women (n=1005)						
n (%)	19 (1.9)	116 (11.5)	425 (42.3)	353 (35.1)	92 (9.2)		
Age (year)*	46.3±6.4	47.0±7.9	46.0±7.0	46.2±7.0	46.5±6.9	0.72^†^	0.89
Current body height (cm)*	155.7±4.5	155.3±5.7	156.6±5.3	156.1±5.4	156.8±5.8	0.22^†^	0.31
Current body weight (kg)*	50.1±5.8	51.1±6.2	52.8±6.6	53.7±7.7	56.5±7.9	<0.001^†^	<0.001
Current BMI (kg/m^2^)*	20.6±1.9	21.2±2.5	21.5±2.6	22.0±2.9	23.0±3.1	<0.001^†^	<0.001
Weight at age 20 (kg)*	47.3±4.5	48.5±5.4	49.5±5.6	50.2±6.8	50.9±7.1	0.01^†^	0.01
BMI at age 20 (kg/m^2^)*	19.5±1.4	20.1±2.1	20.2±2.1	20.6±2.3	20.7±2.5	0.01^†^	0.01
BMI-change (kg/m^2^)*	1.1±2.3	1.1±2.6	1.3±2.7	1.5±3.0	2.3±3.1	0.04^†^	0.07
Physical activity							
≥ 60 min/month	8 (42.1)	54 (46.6)	192 (45.2)	158 (44.8)	39 (42.4)	0.98^‡^	
< 60 min/month	11 (57.9)	62 (53.4)	233 (54.8)	195 (55.2)	53 (57.6)		
Smoking status							
Current smoker	3 (15.8)	8 (6.9)	23 (5.4)	29 (8.2)	10 (10.9)	0.23^‡^	
Former smoker	2 (10.5)	8 (6.9)	16 (3.8)	17 (4.8)	5 (5.4)		
Never smoker	14 (73.7)	100 (86.2)	386 (90.8)	307 (87.0)	77 (83.7)		
Alcohol drinking habit (times/week)*	2.2±2.5	2.0±2.6	1.6±2.3	2.0±2.5	1.9±2.6	0.15^†^	0.60
Energy intake (1000 kcal/day)*	1.78±0.52	1.57±0.49	1.70±0.55	1.62±0.49	1.78±0.78	0.01^†^	0.91

* : mean ± standard deviation

† : one way analyses of variance

‡ : chi-square test

BMI : body mass index

Table 2 shows partial regression coefficients by multiple linear regression analysis in which current BMI was used as the dependent variable. The categories expressing the rate of eating were significantly and positively associated with current BMI independent of daily energy intake both in men and women. In the fully adjusted model, current BMI steadily increased along with the increase in the categorical rate of eating by an increment of -0.99, -0.67, 0.81, and 1.47 kg/m^2^ in ‘very slow’, ‘relatively slow’, ‘relatively fast’, and ‘very fast’ groups, respectively, against the ‘medium’ group in men. In women, the corresponding values were -1.06, -0.35, 0.50, and 1.34 kg/m^2^, respectively.

**Table 2.** Partial regression coefficients (95% confidence intervals) by multiple linear regression analysis against current body mass index.

Independent variables	Model 1*		Model 2*		Model 3^†^	
	Men (n=3737)					
Rate of eating categories						
Very slow	-1.01	(-1.69, -0.34)	-1.01	(-1.68, -0.34)	-0.99	(-1.66, -0.32)
Relatively slow	-0.68	(-0.97, -0.38)	-0.97	(-0.97, -0.37)	-0.67	(-0.97, -0.37)
Medium	0.00	(reference)	0.00	(reference)	0.00	(reference)
Relatively fast	0.83	(0.63, 1.03)	0.81	(0.61, 1.01)	0.81	(0.61, 1.00)
Very fast	1.55	(1.28, 1.82)	1.49	(1.22, 1.76)	1.47	(1.20, 1.75)
Energy intake (1000 kcal/day)	–		0.39	(0.26, 0.51)	0.41	(0.29, 0.54)

	Women (n=1005)					
Rate of eating categories						
Very slow	-0.93	(-2.16, 0.30)	-0.96	(-2.19, 0.27)	-1.06	(-2.29, 0.17)
Relatively slow	-0.39	(-0.94, 0.16)	-0.34	(-0.89, 0.21)	-0.35	(-0.90, 0.20)
Medium	0.00	(reference)	0.00	(reference)	0.00	(reference)
Relatively fast	0.48	(0.10, 0.85)	0.51	(0.13, 0.89)	0.50	(0.13, 0.88)
Very fast	1.42	(0.81, 2.02)	1.39	(0.78, 1.99)	1.34	(0.74, 1.94)
Energy intake (1000 kcal/day)	–		0.39	(0.09, 0.69)	0.43	(0.12, 0.73)

* : adjusted for age

† : adjusted for age, smoking status, physical activity, and alcohol drinking habit

– : not included

The multiple regression analysis in which the BMI at age 20 was regressed by categorical rate of eating, energy intake, age, and lifestyle factors showed that BMI at age 20 gradually increased by -0.59, -0.54, 0.39, and 0.69 kg/m^2^ in ‘very slow’, ‘relatively slow’, ‘relatively fast’, and ‘very fast’ groups, respectively, against the ‘medium’ group in men (Table 3). In women, the corresponding values were -0.80, -0.06, 0.38, and 0.47 kg/m^2^, respectively.

**Table 3.** Partial regression coefficients (95% confidence intervals) by multiple linear regression analysis against body mass index at 20 years of age.

Independent variables	Model 1*		Model 2*		Model 3^†^	
	Men (n=3737)					
Rate of eating categories						
Very slow	-0.57	(-1.14, -0.01)	-0.57	(-1.14, -0.01)	-0.59	(-1.15, -0.03)
Relatively slow	-0.55	(-0.80, -0.30)	-0.55	(-0.80, -0.30)	-0.54	(-0.79, -0.29)
Medium	0.00	(reference)	0.00	(reference)	0.00	(reference)
Relatively fast	0.40	(0.23, 0.57)	0.39	(0.22, 0.55)	0.39	(0.23, 0.56)
Very fast	0.72	(0.49, 0.95)	0.69	(0.46, 0.92)	0.69	(0.46, 0.92)
Energy intake (1000 kcal/day)	–		0.20	(0.09, 0.30)	0.20	(0.10, 0.31)

	Women (n=1005)					
Rate of eating categories						
Very slow	-0.70	(-1.71, 0.32)	-0.70	(-1.71, 0.31)	-0.80	(-1.81, 0.21)
Relatively slow	-0.07	(-0.52, 0.38)	-0.06	(-0.52, 0.39)	-0.06	(-0.51, 0.39)
Medium	0.00	(reference)	0.00	(reference)	0.00	(reference)
Relatively fast	0.38	(0.07, 0.69)	0.39	(0.08, 0.70)	0.38	(0.07, 0.69)
Very fast	0.52	(0.03, 1.02)	0.52	(0.02, 1.02)	0.47	(-0.03, 0.96)
Energy intake (1000 kcal/day)	–		0.06	(-0.19, 0.31)	0.10	(-0.15, 0.35)

* : adjusted for age

† : adjusted for age, smoking status, physical activity, and alcohol drinking habit

– : not included

The partial regression coefficients by multiple linear regression analysis in which BMI-change from age 20 to the current age was used as the dependent variable are shown in Table 4. The association between the rate of eating and BMI-change was similar to that between the rate of eating and current BMI. After full adjustment for energy intake, BMI at age 20, age and lifestyle factors examined, the BMI-change gradually increased along with the increase in the rate of eating by -0.63, -0.34, 0.57, and 1.05 kg/m^2^ in ‘very slow’, ‘relatively slow’, ‘relatively fast’, and ‘very fast’ groups, respectively, against the ‘medium’ group in men. In women, the corresponding values were -0.71, -0.32, 0.34, and 1.14 kg/m^2^, respectively.

**Table 4.** Partial regression coefficients (95% confidence intervals) by multiple linear regression analysis against body mass index (BMI) change from 20 years of age.

Independent variables	Model 1*		Model 2*		Model 3*		Model 4^†^		
	Men (n=3737)								
Rate of eating categories									
Very slow	-0.44	(-1.06, 0.18)	-0.66	(-1.24, -0.09)	-0.67	(-1.24, -0.09)	-0.63	(-1.21, -0.06)
Relatively slow	-0.13	(-0.40, 0.15)	-0.34	(-0.60, -0.08)	-0.34	(-0.59, -0.08)	-0.34	(-0.59, -0.09)
Medium	0.00	(reference)	0.00	(reference)	0.00	(reference)	0.00	(reference)
Relatively fast	0.43	(0.25, 0.62)	0.59	(0.42, 0.76)	0.58	(0.41, 0.75)	0.57	(0.40, 0.74)
Very fast	0.83	(0.58, 1.08)	1.11	(0.88, 1.35)	1.07	(0.84, 1.31)	1.05	(0.82, 1.29)
BMI at age 20 years (kg/m^2^)	–		-0.39	(-0.42, -0.36)	-0.39	(-0.43, -0.36)	-0.39	(-0.42, -0.36)
Energy intake (1000 kcal/day)	–		–		0.27	(0.16, 0.38)	0.29	(0.18, 0.40)

	Women (n=1005)								
Rate of eating categories									
Very slow	-0.23	(-1.51, 1.04)	-0.62	(-1.77, 0.53)	-0.65	(-1.80, 0.49)	-0.71	(-1.86, 0.44)	
Relatively slow	-0.32	(-0.89, 0.25)	-0.36	(-0.87, 0.15)	-0.31	(-0.83, 0.20)	-0.32	(-0.84, 0.19)	
Medium	0.00	(reference)	0.00	(reference)	0.00	(reference)	0.00	(reference)	
Relatively fast	0.09	(-0.30, 0.49)	0.31	(-0.05, 0.66)	0.34	(-0.16, 0.69)	0.34	(-0.15, 0.69)	
Very fast	0.89	(0.27, 1.52)	1.18	(0.62, 1.75)	1.16	(0.59, 1.72)	1.14	(0.58, 1.70)	
BMI at age 20 years (kg/m^2^)	–		-0.56	(-0.63, -0.49)	-0.56	(-0.63, -0.49)	-0.56	(-0.63, -0.49)	
Energy intake (1000 kcal/day)	–		–		0.37	(0.08, 0.65)	0.38	(0.10, 0.67)	

* : adjusted for age

† : adjusted for age, smoking status, physical activity, and alcohol drinking habit

– : not included

Additionally, we performed the same analysis among only non-diabetics (3433 men, 963 women) to eliminate the effect of diabetic medication. The same associations as above were observed in non-diabetics (Table 5).

**Table 5.** Partial regression coefficients (95% confidence intervals) by multiple linear regression analysis against current body mass index (BMI), BMI at 20 years of age, and BMI change from 20 years of age in non-diabetics.

Independent variables	Dedependent variables					

	current BMI*		BMI at age20*		BMI-change*	
	Men (n=3433)					
Rate of eating categories						
Very slow	-0.96	(-1.66, -0.25)	-0.65	(-1.24, -0.07)	-0.55	(-1.15, 0.05)
Relatively slow	-0.67	(-0.97, -0.36)	-0.56	(-0.82, -0.31)	-0.31	(-0.58, -0.05)
Medium	0.00	(reference)	0.00	(reference)	0.00	(reference)
Relatively fast	0.76	(0.55, 0.96)	0.32	(0.15, 0.49)	0.56	(0.38, 0.73)
Very fast	1.46	(1.17, 1.74)	0.64	(0.41, 0.88)	1.05	(0.81, 1.30)
BMI at age 20 years (kg/m^2^)	–		–		-0.38	(-0.41, -0.34)
Energy intake (1000 kcal/day)	0.45	(0.32, 0.59)	0.22	(0.11, 0.34)	0.31	(0.20, 0.43)

	Women (n=963)					
Rate of eating categories						
Very slow	-1.08	(-2.35, 0.18)	-0.6	(-1.63, 0.43)	-0.77	(-1.95, 0.41)
Relatively slow	-0.31	(-0.88, 0.26)	-0.08	(-0.55, 0.38)	-0.28	(-0.81, 0.25)
Medium	0.00	(reference)	0.00	(reference)	0.00	(reference)
Relatively fast	0.55	(0.17, 0.94)	0.42	(0.10, 0.73)	0.37	(0.01, 0.73)
Very fast	1.49	(0.88, 2.11)	0.54	(0.03, 1.05)	1.27	(0.69, 1.85)
BMI at age 20 years (kg/m^2^)	–		–		-0.55	(-0.62, -0.47)
Energy intake (1000 kcal/day)	0.48	(0.16, 0.79)	0.10	(-0.15, 0.36)	0.41	(0.12, 0.71)

* : adjusted for age, smoking status, physical activity, and alcohol drinking habit

– : not included

## DISCUSSION

We showed a positive association between the self-reported rate of eating and current BMI among healthy adult men and women. Moreover, we demonstrated for the first time that the rate of eating was positively associated with BMI at age 20, long-term BMI change from age 20, and height. Our results include some implications about the development of obesity and its prevention.

In our study, current BMI steadily increased along with the increase in the rate of eating in agreement with the previous results,^11^^,^^13^^,^^14^^,^^16^^,^^18^ and the positive association was independent of energy intake. Although most of the previous studies reporting the same association did not consider the energy intake of the subject, only one study concerning the teenaged female college students majoring in dietetic courses by Sasaki et al. reported that after adjusting for energy intake, the mean BMI was higher by 0.5, 1.0, 1.5, and 2.2 kg/m^2^ in the ‘relatively slow’, ‘medium’, ‘relatively fast’, and ‘very fast’ groups, respectively, compared with the ‘very slow’ group in the rate of eating.^16^ Our results were obtained from male and female middle-aged workers, and therefore, may add information to generalize the theory that eating fast leads to obesity.

To our knowledge, this is the first study that examined the association between the current rate of eating, and both the BMI at 20 years of age and the BMI-change from age 20 to the current age. Both BMI at age 20 and the BMI-change gradually increased along with the increase in the current rate of eating. These results give rise to two implications. One is that the person with excess BMI at age 20 or excess BMI gain from age 20 would consequently have fast eating behavior even after middle age. The other is that persons would maintain the fast eating behavior from age 20 or much younger, possibly even from childhood. Many studies report that fast eating was associated with overweight even in childhood.^11^^,^^14^ We also observed the positive association between the rate of eating in adulthood and height. Height is affected by nutrition status in the early years of life and usually stops increasing after puberty. Thus, the positive association may have reflected the larger growing of fast eater before adulthood. When these results are taken together, it can be surmised that an eating behavior such as fast eating acquired in childhood may be maintained up to and during adulthood.

One mechanism underlying the relationship between the rate of eating and obesity would be the increase in energy intake in the fast eater because energy intake per day increased significantly with the increase in the rate of eating. Sakata and Yoshimatsu suggested, from their study of obese rat, that an eating-rate abnormality might be the result of a defect in hypothalamic neuronal histamine.^20^ Due to a lack of satiety, rapid eating may cause overeating before the stomach senses fullness. This is consistent with the positive association of the rate of eating and energy intake observed in our study.

Other mechanisms remain to be elucidated and are beyond the scope of this study. However, there is a report implicating blood insulin and the glucose response related with the rate of eating. Jenkins et al. demonstrated the metabolic benefits that might result from reducing the rate of nutrient delivery in their experiment in which nine healthy volunteers took 50 g glucose in 700 mL water on two occasions; one, over 5-10 min (bolus ingestion) and another, at a constant rate over 3.5 hr (sipping ingestion).^21^ Despite similar 4-hr blood glucose areas, a larger increase was seen in the serum insulin level after the bolus ingestion. The glucose disappearance rate, serum free fatty acid levels, and branched-chain amino acid, all indicated a decrease in insulin sensitivity after bolus ingestion compared with sipping.

Although they used soluble foods, we assumed that the same insulin response would be induced by eating usual meal containing solid foods rapidly, and that repetition of rapid eating every day would lead to an intermittent state of decreased insulin sensitivity, in other words, increased insulin resistance. Boyko et al. followed 137 non-diabetic Japanese-American men for changes in visceral adiposity over 5 years and demonstrated that insulin resistance of the stage accompanied with high insulin level preceded increased visceral adiposity.^22^ Judging from these reports, it is suggested that the repetition of rapid eating would cause obesity through insulin resistance.^23^^,^^24^

Additionally, other unknown factors might mediate the relationship between fast eating and obesity; for example, mental stress might be a confounding factor. Mental stress or psychological workload has been reportedly associated with obesity.^25^^-^^28^ If busy workers under mental stress cannot help eating fast owing to the limited time for eating, a positive association may develop between rapid eating and obesity.

There are several limitations in our study. First, we used a self-reported rate of eating, of which the validity may influence the observed results. According to Sasaki et al., a high level of concordance has been reported between self-reported and friend-reported rate of eating,^16^ though their subjects were 18-year-old students, and their ages were different from those in the present study. Further studies are needed to verify the validity of self-reported rate of eating objectively. Second, we do not have information either on the rate of eating at age 20 or the data relating transition of eating rates over long periods. Our data are cross-sectional and therefore do not prove a causative role for the rate of eating in the increase of weight. Third, we used self-reported weight at age 20, which might be biased. As for the validity of long-term recall of past body weight, however, we reported previously that self-reported weight at age 20 was a reliable measure for use in correlation analysis among adults aged 34 to 61.^29^ Fourth, we estimated energy intake using a self-administered dietary assessment questionnaire, i.e., BDHQ.^19^ The validity against 16-day energy intake assessed with semi-weighed dietary record was 0.24 and 0.26 in men and women, respectively (unpublished data, personal communication by Sasaki S, 2005). Therefore, the inclusion of energy intake into the present analysis may be not necessarily sufficient for its adjustment. Fifth, the positive association between rate of eating and BMI might be due in part to other characteristics of lifestyle or eating behavior which we could not consider. Finally, the subjects in this study comprised apparently middle-aged Japanese men and women, and thus the results may not be applicable to Westerners with a higher BMI.

We discussed our results from the viewpoint of preventing obesity and now believe that a slower rate of eating would contribute to losing weight, though we do not understand whether longer meal times or increased chewing would be more effective. Spiegel et al.^13^ assessed the changes in eating behavior including slower rate of eating in obese women in a behavioral weight control program. They observed that longer mealtime was associated with greater weight loss.^13^ However, a slower rate of eating was not maintained during the behavioral weight control program, suggesting that therapeutic efforts should be directed at the maintenance of behavior changes. We also showed in our study that the fast eater tended to be younger than the slow eater in males. This may imply that fast eating behavior they have spread among young Japanese, indicating the importance of acquiring correct eating behavior earlier in life.

In conclusion, we found not only a statistically positive association between the rate of eating and energy intake, and current BMI, but also positive associations with previous BMI at age 20, long-term BMI-change from age 20 and height among Japanese adult men and women. In addition, the same associations as above were observed independently of energy intake. Our results thus strongly suggest that the rate of eating may be an important factor for the prevention of obesity in all generations.
